# New agents in the Treatment of Myeloma Bone Disease

**DOI:** 10.1007/s00223-017-0351-7

**Published:** 2017-11-02

**Authors:** Elizabeth S. Ring, Michelle A. Lawson, John A. Snowden, Ingrid Jolley, Andrew D. Chantry

**Affiliations:** 1 Department of Oncology and Metabolism, Faculty of Medicine, Dentistry and Health, The University of Sheffield Medical School, Beech Hill Road, Sheffield, South Yorkshire S10 2RX UK; 2 Sheffield Myeloma Research Team, Department of Oncology and Metabolism, Mellanby Bone Centre, School of Medicine and Biomedical Sciences, University of Sheffield, Beech Hill Road, Sheffield, S10 2RX UK; 3 Department of Haematology, Sheffield Teaching Hospitals NHS Foundation Trust, Sheffield, UK; 4 Department of Radiology, Sheffield Teaching Hospitals NHS Foundation Trust, Sheffield, UK

**Keywords:** Myeloma, Myeloma bone disease, Bone remodelling, Antiresorptive agents, Anabolic agents

## Abstract

Patients with multiple myeloma develop a devastating bone disease driven by the uncoupling of bone remodelling, excess osteoclastic bone resorption and diminished osteoblastic bone formation. The bone phenotype is typified by focal osteolytic lesions leading to pathological fractures, hypercalcaemia and other catastrophic bone events such as spinal cord compression. This causes bone pain, impaired functional status, decreased quality of life and increased mortality. Early in the disease, malignant plasma cells occupy a niche environment that encompasses their interaction with other key cellular components of the bone marrow microenvironment. Through these interactions, osteoclast-activating factors and osteoblast inhibitory factors are produced, which together uncouple the dynamic process of bone remodelling, leading to net bone loss and focal osteolytic lesions. Current management includes antiresorptive therapies, i.e. bisphosphonates, palliative support and orthopaedic interventions. Bisphosphonates are the mainstay of treatment for myeloma bone disease (MBD), but are only partially effective and do have some significant disadvantages; for example, they do not lead to the repair of existing bone destruction. Thus, newer agents to prevent bone destruction and also promote bone formation and repair existing lesions are warranted. This review summarises novel ways that MBD is being therapeutically targeted.

## Introduction

Myeloma bone disease (MBD) is a hallmark feature of multiple myeloma (MM). MM is a cancer of differentiated B lymphocytes, known as plasma cells, involving their clonal proliferation in the bone marrow. It is characterised by the production of monoclonal immunoglobulins (known as a paraprotein, monoclone or M-spike) and by the uncoupling of the dynamic process of bone remodelling [1]. MM accounts for 1% of new cancers worldwide, is the second most common haematological malignancy and has a 5-year survival rate of 49% [2, 3].

**Fig. 1:**
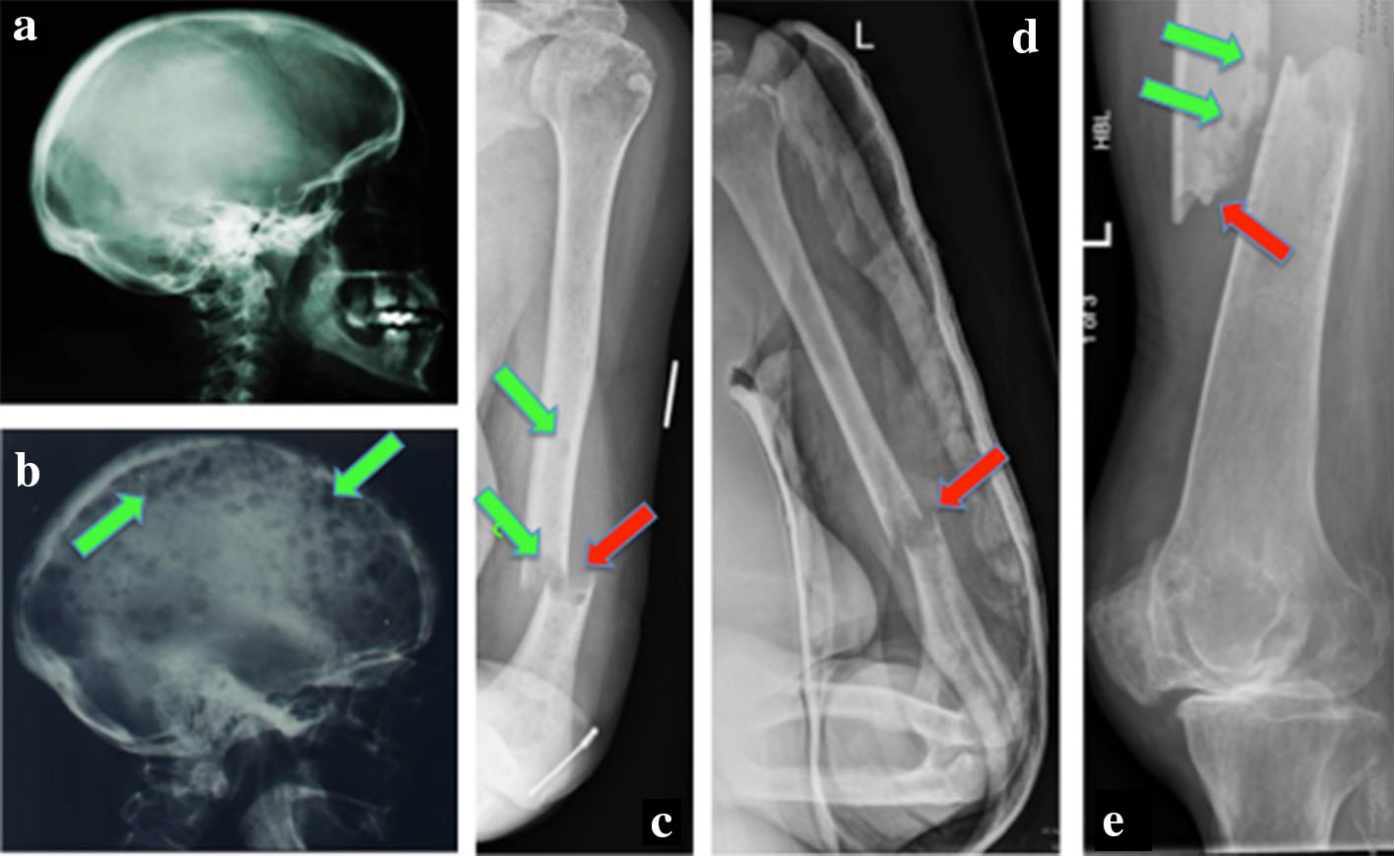
X-ray examples of serious but preventable myeloma-induced osteolytic lesions and pathological fractures potentially preventable if detected earlier. **a** Normal skull. **b** Myeloma ‘pepper pot skull’ riddles with lytic lesions. **c**, **d** Pathological fractures through lytic lesions in the distal shaft of the left humerus. **e** Pathological fracture through the proximal shaft of the left femur

MM is a debilitating disease with features including hypercalcaemia, renal impairment, anaemia and bone disease (summarised in the mnemonic CRAB) [4]. In MM, 80–90% of patients develop MBD (Fig. 1), leading to pathological fractures, spinal cord compression and pain, collectively referred to as skeletal-related events (SREs), which contribute to a reduced quality of life [5]. Although there has been a substantial increase in overall survival (OS) in the past 10 years, 85% of osteolytic lesions develop during management, which highlights a key pitfall in the current management MBD [6, 7].

MBD occurs due to the interactions between malignant plasma cells (MPCs) and cells in the bone marrow microenvironment (BMME), leading to accelerated overall bone loss and the formation of focal osteolytic lesions. Normal bone modelling is dysregulated leading to the uncoupling of osteoclast and osteoblast activity, excessive osteoclastic bone resorption and substantially reduced osteoblastic bone formation [1, 3]. Furthermore, anti-MM treatments, such as dexamethasone, can induce further bone loss, potentiating MBD. Current treatments aim to prevent further myeloma-induced bone disease through the use of antiresorptive therapy. Recently, a number of potential bone anabolic agents have been assessed in preclinical models of MM and other novel agents are being developed as our understanding of MBD improves [8–10]. This review focuses on current and novel agents that specifically target MBD.

## Pathophysiology of Myeloma Bone Disease

Under normal physiological conditions, osteoblasts and osteoclasts work effectively in unison to remodel bone via bone formation and bone resorption, respectively [1, 5]. Over the course of 7 years, the entire skeleton can be remineralised and adapt to physiological stress due to the opposing actions of osteoblasts and osteoclasts [5]. Osteoclasts and osteoblasts are the main cells involved in bone modelling; however, this process is facilitated by osteocytes, cytokines and hormones [1].

Osteoclasts originate from monocytes and digest the bone matrix through the secretion of enzymes [11]. Osteoblasts differentiate from mesenchymal stem cells and create the bone matrix through the secretion of collagen [12]. Furthermore, immature osteoblasts secrete cytokines such as interleukin-6 (IL-6) to upregulate osteoclasts and mature osteoblasts secrete osteoprotegerin (OPG) to inhibit the activation of osteoclasts [4, 13]. As new bone is formed, osteoblasts become trapped and differentiate into osteocytes [11]. Osteocytes contribute factors, such as sclerostin, to both osteoclastogenesis and osteoblastogenesis to control bone remodelling.

MPCs cause the uncoupling of this bone remodelling process by interacting with the BMME and stromal components to induce osteoclast-activating factors (OAFs), first described by Mundy et al., to promote osteoclastogenesis [6, 14]. In the initial stages of the disease, both osteoblasts and osteoclasts are recruited to initiate bone resorption. Myeloma cells produce IL-1 and TNF, which stimulate osteoblast progenitor cells to differentiate into osteoblasts, thus recruiting more osteoblasts to the site. Osteoblasts secrete IL-6, which is a potent myeloma growth factor and promoter of osteoclastogenesis [4, 15].

**Fig. 2:**
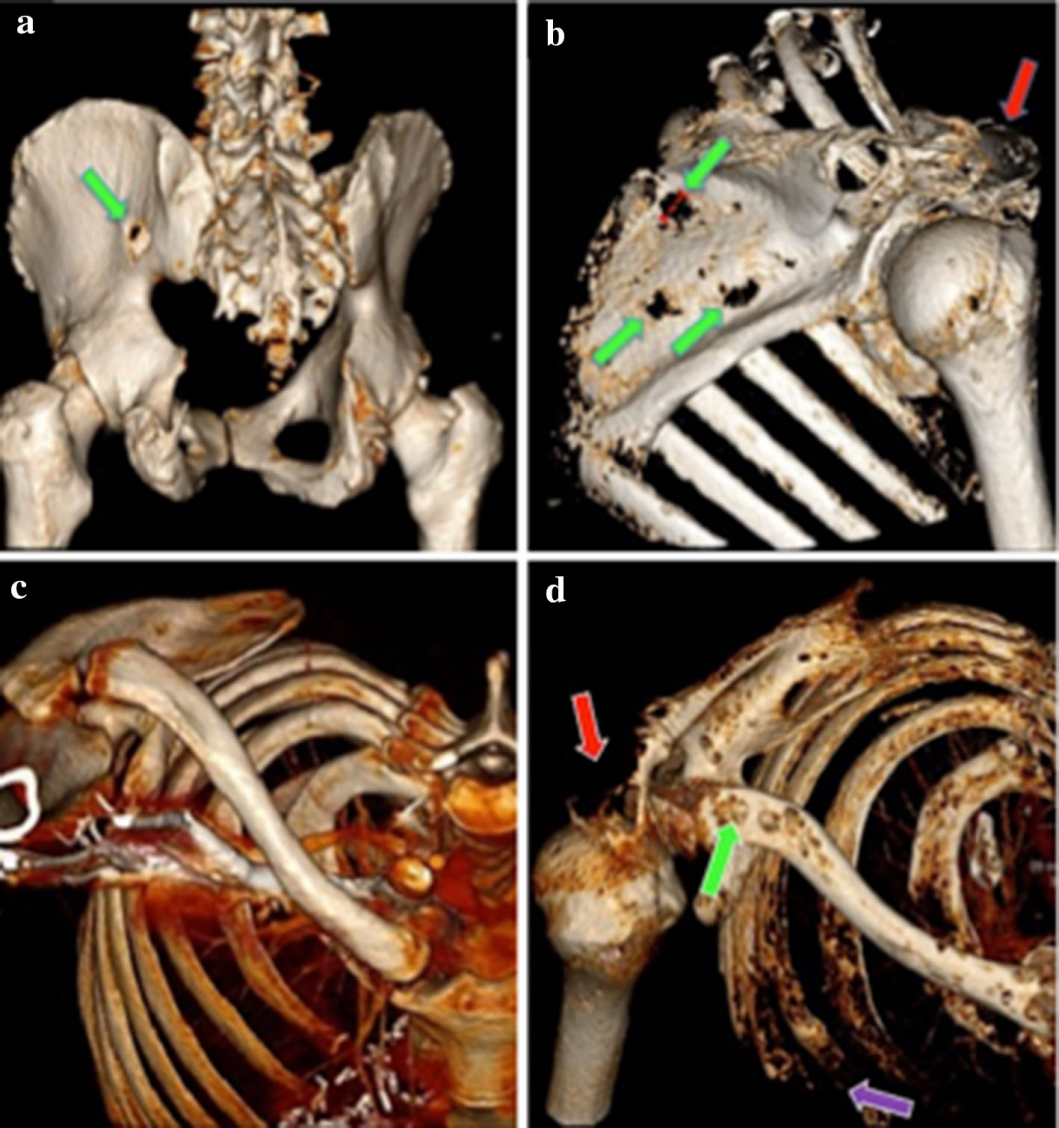
3D reconstructions of computerised tomography (CT) images using standard diagnostic settings demonstrating two patients with widespread myeloma-induced bone disease, leading to potential serious consequences. **a** Lytic lesion penetrating through the ischium (green arrow). **b** Multiple lytic lesions throughout the scapula (green arrows) with the acromion completely destroyed by myeloma bone disease (red arrow). **c** Example of normal bone from the shoulder, clavicle and ribs. **d** Contrast image of the patient riddled with lytic lesions due to myeloma bone disease. The acromion process is destroyed (red arrow), multiple lytic lesions are present throughout the clavicle (green arrow) and the anterior ribs have been destroyed (purple arrow) (Color figure online)

However, once MBD is established, osteoblasts decrease in number [15]. The mechanism that initiates this still remains unclear; however, this possibly is achieved through the release of osteoblast inhibitory factors (OBIs), as described by Bataille et al. [4, 16]. Along with inhibiting bone formation, a further reason osteoblasts are hypothesised to be inhibited is due to decorin, a small leucine-rich proteoglycan, which is produced by osteoblasts. Li et al. [17] demonstrated that decorin has an anti-myeloma effect through inhibiting transforming growth factor beta (TGF-β) and decreasing tumour growth. However, there is conflicting evidence as to whether decorin is related to the development of osteolytic lesions [18, 19]. Furthermore, myeloma cells induce aberrant changes in osteoprogenitors, through alterations in microRNA, which prevents their differentiation to osteoblasts, thus reducing the number of osteoblasts further [20]. With the suppression of osteoblastogenesis and the hyperactivation of osteoclasts, the formation of osteolytic lesions expands from a singular site (Fig. 2), to invade the entire bone marrow and destroy the surrounding bone, eventually spreading into the blood and metastasising to other bone sites [11].

## Osteoclastic Bone Resorption is Increased in Myeloma

The balance between osteoblasts and osteoclasts is maintained through the ratio of OPG:receptor activator of nuclear factor kappa B (RANK) [21]. RANK and its ligand (RANKL) activate the downstream nuclear factor kappa B (NF-kB), which subsequently activates osteoclast precursors and causes their differentiation to mature osteoclasts, whilst simultaneously decreasing osteoclast apoptosis [3, 6]. OPG is a soluble decoy receptor that inhibits RANK via mimicking RANKL, in order to increase osteoblast activity and promote bone formation [5, 6, 22].

Increased bone resorption is achieved through the uncoupling of OPG:RANK:RANKL and an increased production of RANKL [23]. MPCs adhere to bone marrow stromal cells (BMSCs), which increases the production of OAFs, such as RANKL, IL-6 and Activin A [1]. IL-6 is a cytokine that is highly elevated in MM and shown to correlate with increased bone destruction [24]. BMSCs and macrophages are the main sources of IL-6, promote osteoclastogenesis, increase MPC population and prevent apoptosis through the induction of the P13k/AKT pathways allowing proliferation and survival of MPCs [3, 5, 25].

Macrophage inflammatory protein-1 alpha (MIP-1α) is secreted by MPCs and causes osteoclastogenesis through binding to chemokine receptor type 1 (CCR1) and chemokine receptor type 5 (CCR5) on osteoclasts [3, 26]. Simultaneously, they improve the adhesion between MPCs and BMSCs, therefore promoting a further increased production of IL-6 and RANKL. Finally, MPCs create a feedback loop, to ensure their own survival by producing MIP-1α, which induces pathways such as mitogen-activated protein (MAPK) pathway [5].

OAFs that are elevated in MM patients include IL-3, which increases osteoclast activity in combination with RANKL and MIP-1α and synergistically works with IL-6 to promote MPC growth [1, 27]. Vascular endothelial growth factor (VEGF), a signalling protein, and osteopontin, a non-collagenous protein, are increased in MM and both increase angiogenesis and osteoclastogenesis [1, 14, 23]. Tanaka et al. demonstrated that when both VEGF and osteopontin were inhibited, angiogenesis and bone resorption were significantly reduced, highlighting their potential role in MBD [28].

## Inhibition of Osteoblastic Bone Formation is seen in Myeloma

MBD is enhanced further by osteoblastic inhibition, resulting in bone loss with no repair. A key pathway linked to osteoblast differentiation, highlighted by Day et al. [12], is the canonical Wnt pathway. β-Catenin, the downstream product of the Wnt pathway is a potent promoter of OPG and osteoblastogenesis [29]. Wnt proteins bind to a cell surface receptor complex consisting of Frizzled and lipoprotein-related (Lrp) 5/6 proteins [5]. This activates a downstream cascade, which prevents the degradation of β-catenin.

Levels of dickkopf-1 (Dkk-1) produced by both BMSCs and MPCs are increased in the serum and the bone marrow milieu of MM patients inhibiting the Wnt pathway, resulting in a decrease in osteoblastogenesis [9, 26, 30]. Dkk-1 further inhibits immature osteoblasts to enable the maximum amount of IL-6 to be secreted [13]. Secreted frizzled-related protein 2 (sFRP-2), a further Wnt antagonist, preventing the binding of Wnt to Frizzled, is found to be overexpressed in MM patients [5].

The transcription factor runt-related transcription factor 2 (Runx2)/core-binding factor runt domain alpha subunit 1 (CBFA1) is a key driver in osteoblast differentiation [31]. Runx2/CBFA1 works together along with other transcription factors such as osterix to induce bone formation [32]. MPCs have the ability to inhibit Runx2/CBF1A, therefore downregulating the differentiation of osteoblast from osteoprogenitor cells and causing an increase in osteolytic lesions [1, 33]. Furthermore, Runx2/CBFA1 mediates the secretion of OPG and, therefore, upon inhibition decreases OPG and increases osteoclastogenesis [33].

Development of osteolytic lesions is stimulated further by a vicious cycle involving several other factors. TGF-β is produced by the bone matrix during bone resorption and inhibits osteoblast differentiation [3]. OAFs such as IL-3 and IL-7 play a dual role, by also inhibiting osteoblasts via inducing Activin A and suppressing Runx2, respectively [34]. Furthermore, MPCs secrete hepatocyte growth factor (HGF), which inhibits bone morphogenetic proteins (BMPs) and suppresses runx2, therefore inhibiting osteoblastogenesis [35].

**Fig. 3:**
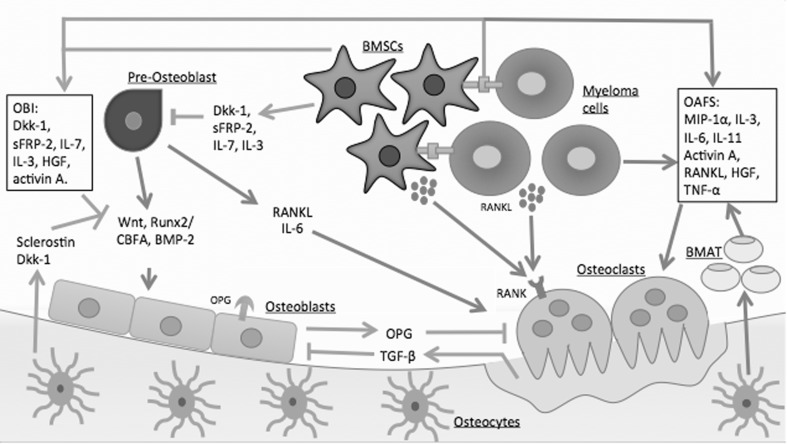
Pathophysiology of MBD. The uncoupling of osteoclasts and osteoblasts is stimulated by the release of osteoclast-activating factors (OAFs) and osteoblast inhibitory factors (OBIs). These factors are released by the adhesion of bone marrow stromal cells (BMSCs) to myeloma cells causing upregulation of osteoclast and bone resorption, whilst simultaneously inhibiting osteoblasts and bone formation. Osteocytes also play an important role by releasing sclerostin, which inhibits osteoblast differentiation and increases bone marrow adipose tissue (BMAT). *Dkk-1* dickkopf-1, *sFRP-2* secreted frizzled-related protein 2, *IL-7* interleukin-7, *IL-3* interleukin-3, *HGF* hepatocyte growth factor, *Runx2* runt-related transcription factor 2, *CBFA* core-binding factor alpha, *BMP-2* bone morphogenetic protein 2, *RANK* receptor activator of nuclear factor kappa B, *RANKL* receptor activator of nuclear factor kappa B ligand, *IL-6* interleukin-6, *MIP-1α* macrophage inflammatory protein-1 alpha, *OPG* osteoprotegerin, *TGF-β* transforming growth factor beta, *TNF-α* tumour necrosis factor alpha

Tumour necrosis factor α (TNF-α) also plays a dual role in both osteoclastogenesis and inhibition of osteoblast differentiation. MPCs induce high levels of TNF-α in the marrow microenvironment [36]. TNF-α increases BMSC production of OAFs such as RANKL and IL-6 through increasing the transcription factor spliced X-box binding protein 1, thus increasing osteoclastogenesis [37]. TNF-α inhibits osteoblast differentiation by decreasing runx2 and osterix, which are key regulators in osteoblast differentiation [38]. Furthermore, TNF-α can induce apoptosis of mature osteoblasts [39]. Thus, the development of MBD correlates directly to the stimulation of osteoclasts and inhibition of osteoblasts (Fig. 3).

Furthermore, anti-MM treatment can exacerbate bone loss and contribute to MBD [40]. High-dose steroids such as dexamethasone and prednisolone are commonly used in MM, to reduce inflammation, improve the patients’ immune system and reduce the side effects of chemotherapy [41]. Steroids inhibit IL-6 and reduce NF-kB, inducing apoptosis in MPCs, and thus provide a backbone to many MM treatment regimes [42]. However, high-dose dexamethasone is also known to inhibit osteoblastogenesis, downregulate OPG and in turn upregulate the interaction between RANK and RANKL, thus promoting osteoclastogenesis and bone resorption [41]. This highlights the clinical challenge of prescribing a dose of high-dose steroids that positively impacts MM but without causing progression of MBD. In recent studies, combining steroids such as dexamethasone with immunomodulatory drugs and bisphosphonates (inhibit bone resorption) has reduced the extent of the bone loss caused by high-dose steroids [43].

## Osteocytes Regulate Bone Remodelling in MBD

Osteocytes are the most abundant bone cells, making up 95% of all bone cells [44]. Osteocytes contribute to the vicious cycle of MBD by regulating bone remodelling through releasing paracrine factors, such as sclerostin and RANKL that affect osteoblasts and osteoclasts, respectively. Giuliani et al. demonstrated that MM patients with MBD had fewer osteocytes than healthy controls, indicating that osteocyte apoptosis may play a role in the development of osteolytic lesions [45]. Osteocyte apoptosis is accompanied by increases in RANKL, therefore promoting osteoclast differentiation and regulating bone resorption [45]. Furthermore, MPCs caused the upregulation of OAF IL-11 from osteocytes, promoting osteoclast differentiation [34].

Osteocytes secrete Dkk-1 and sclerostin, a potent inhibitor of bone formation [30]. Sclerostin inhibits the canonical Wnt pathway, therefore downregulating the production of Wnt target genes, such as OPG, and increasing the RANKL/OPG ratio, leading to an inhibition in osteoblast differentiation and bone formation [46]. Furthermore, osteocytes are able to create a network of interactions from cell-to-cell contact between each other to cells on the cell surface and are able to distribute cytokines throughout the bone marrow, thus making osteocytes the central regulators of bone homeostasis and highlighting how osteocytes may therefore play an important role in the development of MBD [44].

## Current Treatment of MBD

**Fig. 4:**
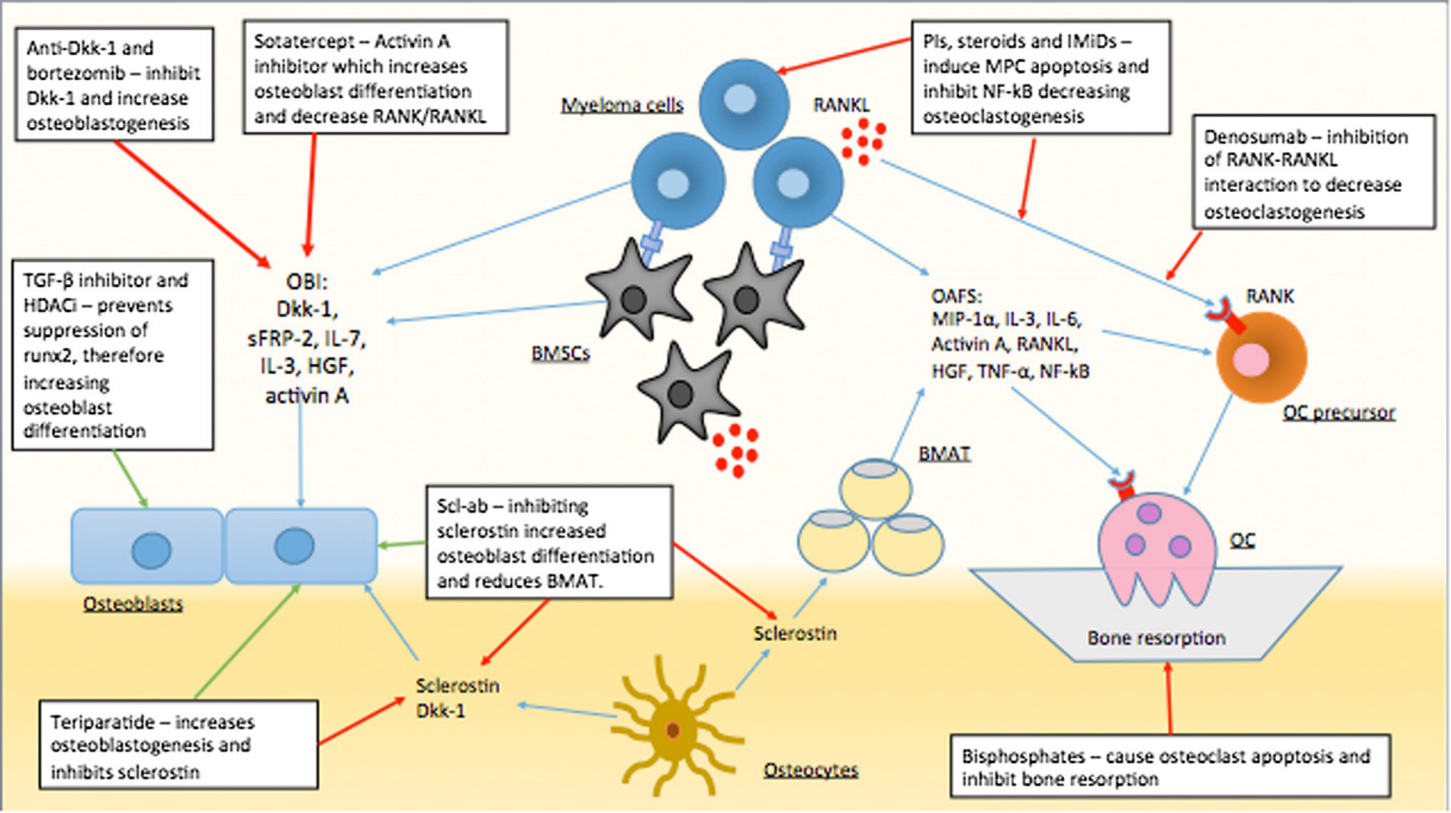
MBD treatments and their interactions in the BMME. MBD treatments use multiple different mechanisms in order to reduce bone resorption and increase bone formation to repair osteolytic lesions. A plethora of treatments are currently in trials; however, a combination of both anabolic and antiresorptive methods appears to have the most potential for healing MBD. *RANKL* receptor activator of nuclear factor kappa B ligand, *RANK* receptor activator of nuclear factor kappa B, *PIs* proteasome inhibitors, *IMiDs* immunomodulatory agents, *OC* osteoclast, *Scl-ab* anti-sclerostin antibody, *Dkk-1* dickkopf-1, *sFRP-2* secreted frizzled-related protein 2, *IL-7* interleukin-7, *IL-3* interleukin-3, *HGF* hepatocyte growth factor, *Runx2* runt-related transcription factor 2, *TGF-β* transforming growth factor beta, *NF-kB* nuclear factor kappa B, *BMSCs* bone marrow stromal cells, *BMAT* bone marrow adipose tissue, *OBIs* osteoblast inhibitory factors, *OAFs* osteoblast-activating factors

Once MM has been diagnosed and MBD is detected, various treatments are available. A multidisciplinary approach is needed to ensure that a patient’s quality of life is maintained through the use of analgesia for pain, surgery or radiotherapy for MBD. MBD will progress without adequate anti-MM treatment, and thus a patient management plan needs to treat the underlying MM through the use of anti-MM treatment and combine this with MBD treatment. Preventative therapies are needed to delay disease progression in MBD, with the mainstay of treatment being antiresorptive agents. Bisphosphonates are the only treatment licensed for the prevention of MBD worldwide. However, they do not completely prevent osteolytic lesions and fail to promote new bone formation or repair of existing lesions [47]. Recently, novel anabolic agents such as anti-sclerostin and anti-Dkk1, which promote osteoblastogenesis and bone formation and have the potential to repair existing lesions, have been developed, which may lead to a substantial improvement of MBD (Fig. 4) [9, 10, 30].

## Antiresorptive Therapies

### Bisphosphonates (BPs)

The initial first-line treatment for MBD is antiresorptive therapies, such as BPs. These originate from a key observation made by Fleisch and Neuman that body fluids, such as urine, contain natural inhibitors of calcification [48]. This compound was found to be inorganic pyrophosphate (PPi). Further studies revealed that high levels of PPi cause defective skeletal mineralisation, whilst low levels caused excessive mineralisation and bone formation [49]. This led to the development of different PPi analogues to inhibit abnormal calcification, eventually producing BP analogues (P–C–P motif). Although initially used to prevent calcification of soft tissues, BPs were soon discovered to inhibit bone resorption, thus marking the beginning of the era of their use as antiresorptives [50, 51].

Non-nitrogen-containing BPs, such as clodronate, are thought to induce apoptosis of osteoclasts by causing the accumulation of non-hydrolyzable ATP analogues [52]. Nitrogen-containing BPs, such as pamidronate and zoledronic acid, bind to hydroxyapatite and then cause osteoclast apoptosis via inhibition of the mevalonate pathway via the enzyme farnesyl diphosphate synthase [1, 53, 54].

The nitrogen-containing BPs, such as zoledronic acid, have proved to be significantly superior at decreasing SREs than the non-nitrogen-containing BPs, such as clodronate, which was highlighted in the MRC Myeloma IX trial [47]. A subset analysis of the MRC Myeloma IX trial also demonstrated a significant reduction in tumour burden in patients receiving zoledronic acid compared to patients receiving clodronate. Although the mechanism of this antitumour effect is uncertain, this finding has provided strong additional rationale for the use of zoledronic acid rather than clodronate in the treatment of patients with MM. Comparatively, the choice for zoledronic acid to be used as the first-line treatment instead of pamidronate is also due to reduced infusion time and reduction in other adverse events [54–56].

Despite BPs being the initial treatment of choice, the longevity of their use is limited due to their side effects. These include renal toxicity requiring dose reduction in patients with renal impairment, flu-like symptoms and gastrointestinal upset during administration, atrial fibrillation, atypical femoral fracture and osteonecrosis of the jaw (ONJ), which can occur in 3.5% of patients [47]. Although inferior, nitrogen-containing BPs, such as clodronate, exhibit a lower rate of ONJ compared to zoledronic acid (1 vs. 4%, respectively) [47]. Furthermore, pamidronate can be administered to patients with significant renal impairment [57]. Due to these risks, BPs are recommended for up to 2 years before a break in treatment and the continuation to be administered at much longer intervals.

### Denosumab

Denosumab is an anti-RANKL monoclonal antibody, designed to prevent osteoclast function and osteoclastogenesis by preventing the RANK–RANKL interaction [5]. Denosumab thus mimics OPG by decreasing the amount of RANKL available. Currently, denosumab is not approved for use in MM. However, there is an ongoing clinical trial (NCT00330759) comparing denosumab to zoledronic acid in MM patients, the preliminary results of which show that denosumab has similar results for time to future skeletal events, but has significantly lower renal toxicities compared to zoledronic acid (10 vs. 17.1%) [58].

Although there are limited studies primarily aimed at denosumab and MM, those that have reported data have concluded that denosumab is non-inferior to zoledronic acid. Henry et al. [59] showed no significant difference between the two arms in regards to delaying first onset SRE, OS and progression-free survival (PFS). However, denosumab did exhibit higher rates of hypocalcaemia and similar levels of ONJ, but had potentially higher mortality rates. This study did conclude that MM needed to be investigated further as their results were for a variety of cancers and that there was a possible variant in heterogeneity of the population used.

Raje et al. [60] found similar findings in a subset of MM patients and concluded that denosumab was non-inferior. However, they did raise the concern of a higher mortality in the denosumab arm compared to zoledronic acid (22 vs. 9%). This study had a number of confounding factors including a small subgroup of patients from a larger trial and a large amount of withdrawals with no follow-up which may have skewed the results towards zoledronic acid.

Denosumab is recommended when BPs cannot be prescribed, for example due to renal toxicities. There is also a recommendation to use denosumab if hypercalcaemia of malignancy occurs and is refractory to BPs [61]. Denosumab is not nephrotoxic and can be given as a subcutaneous injection, which allows easier access for patients to this treatment and provides a potential alternative to those that cannot have BPs.

## Anabolic Agents

### Parathyroid Hormone

Parathyroid hormone (PTH) has been shown to have anabolic affects in bone remodelling in osteoporosis. At high levels, PTH causes an increase in bone resorption due the release of calcium from the bone initiated by PTH [62, 63]. However, intermittent doses have been shown to be anabolic in nature rather than resorptive. Teriparatide, a recombinant form of PTH, has been approved for use in women with osteoporosis [64]. The mechanism for teriparatide’s anabolic effect is unclear, but it is thought to be due to PTH having a direct effect on osteoblasts, therefore increasing osteoblastogenesis and also inhibiting sclerostin, a potent promoter of osteoclastogenesis [62].

Pennisi et al. [65] studied PTH administration in mouse models for MM and showed that there was an increase in bone mineral density via the upregulation of osteoblasts, although this was not seen in vitro. However, the myeloma cell line that was used did not express PTH receptors. In addition, teriparatide has been shown to improve bisphosphate-associated ONJ after alendronate was stopped, by showing significant healing of necrotic bone in various patient case reports, showing a potential therapeutic use in combination with BPs [66].

However, in contradicting studies, high levels of PTH may be a potential risk factor for MM. Kang et al. [63] demonstrated that high PTH levels may facilitate the growth of myeloma cells via secretion of IL-6 and that higher PTH levels at diagnosis correlated with a poorer PFS but no difference in OS. Furthermore, in certain cancers such as prostate cancer, PTH may increase metastases [67]. The safety and efficacy of PTH in MM are therefore still to be established, but warrant further enquiry given promising results obtained in patients with osteoporosis.

### Anti-Dkk-1

Dkk-1 is a potent regulator of the Wnt signalling pathway and inhibits the Frizzled co-receptor LRP6 [68]. Dkk-1 is produced by BMSCs and MPCs and it has been found to be elevated in MM patients. Dkk-1, along with sclerostin, decreases the levels of β-catenin, which in turn reduces osteoblast differentiation [69]. If osteoblasts cannot repair the osteolytic lesions, even with the use of antiresorptive agents to prevent bone resorption, MBD will persist.

Tian et al. [9] first hypothesised that there is an increase in Dkk-1 in MPCs and the bone marrow of MM patients. They showed that Dkk-1 inhibits the differentiation of osteoblasts and increases the activity of osteoclasts via increased expression of RANKL from osteocytes. This study used patients with varying penetrance of MM, demonstrating an increase in Dkk-1 in those with active MBD, as well as those without osteolytic lesions.

Anti-Dkk-1 agents have been investigated as a novel target, including the agent BHQ880, a humanised IgG anti-Dkk-1 monoclonal antibody. In vitro and in vivo analyses of the effects of BHQ880 were highlighted by Fulciniti et al. [24], showing that BHQ880 was successful at inhibiting Dkk-1 and increasing osteoblast differentiation and activity, as shown by the increase in trabecular thickness. BHQ880 activity in vivo was analysed by H&E staining of the bone to highlight the amount of myeloma cells and was monitored by IL-6 murine blood levels, which are produced from BMSCs and decrease when these differentiate into osteoblasts, indicating that a higher level of IL-6 correlates with a decrease in osteoblast differentiation. However, a limitation of this method is that IL-6 is not just produced by myeloma cells so this may not be the most accurate way to monitor BHQ880 activity.

Finally, they concluded an unknown effect of BHQ880 on osteoclastogenesis, implying that this would be used as a combination treatment with antiresorptive agents. A limitation of this study was that only one cell line was used in the in vivo models, which may not be representative of MM.

A phase 1b multicentre study has been undertaken by Iyer et al. [70], which combined BHQ880 with zoledronic acid and an anti-myeloma treatment regimen. They reported that this combination was well tolerated by MM patients and caused a delay in SREs whilst increasing bone density. However, these results are from the combined treatment, making it unclear how much BHQ880 had an independent effect on these outcomes.

A further mechanism for anti-Dkk-1 treatment that has recently been highlighted is through the interaction of Dkk-1 and microRNA (mi-RNA). Mi-RNA contributes to cell proliferation, apoptosis and differentiation, and the downregulation of several mi-RNA can lead to tumour progression. Xu et al. [71] demonstrated that mi-RNA152 directly targeted Dkk-1 and reduced the expression of Dkk-1. Mice were injected into the femur with myeloma cells (MM.1S) that were infected with mi-RNA152. This caused an elevation in mi-RNA152, which sequentially decreased the expression of Dkk-1, resulting in decreased bone destruction and increased bone mineralisation. Limitations of this study include using only one cell line for analysing osteolytic lesions and that by manipulating mi-RNA this could lead to unwanted systemic effects.

A phase II clinical trial has been completed (NCT01337752), which evaluates the use of BHQ880 when BPs are contradicted due to renal insufficiency. The results of this trial are yet to be published, however once available these results may highlight BHQ880 use in MM. However, anti-Dkk-1 treatment still needs thorough investigation to determine its optimal use in MBD. A concern with this treatment is that some patients do not have increased levels of Dkk-1 and in end-stage disease Dkk-1 decreases [9]. This may be due to the increased interaction of MPCs with osteoclasts or due to a mutation in p53 which is strongly associated with Dkk-1. However, follow-up research would be needed to ensure that inhibiting Dkk-1 did not advance the disease [11].

### Anti-sclerostin

Sclerostin, encoded by the SOST gene, is produced by osteocytes, binds to Wnt co-receptors LRP5/6 and antagonises the pathway [72]. Sclerostin has been shown to be an important mechanism in osteoporosis; however, its importance has not been established in MBD [69]. Romosozumab, a humanised monoclonal anti-sclerostin antibody, has been approved in osteoporosis, shows marked improvement in bone formation and bone mineral density, whilst decreasing bone resorption markers, and could be a potential agent for MBD [73]. However, Amgen have recently released a statement regarding their ARCH study that romosozumab increases a patient’s cardiovascular risk by 2.5% compared to alendronate (1.9%), causing the European Medicine Agency to rule that romosozumab be used only in patients with no history of cardiac problems.

MM upregulates SOST and increases the expression of sclerostin from osteocytes. Delgado-Calle et al. [74] showed in mice with MM a raised level of sclerostin and a decrease in OPG of 50%. This correlated with a decrease in osteoblast markers, providing evidence for a link between the inhibition of the Wnt signalling pathway and osteoblast differentiation in the presence of raised sclerostin.

Reagan et al. [10] demonstrated in vivo that anti-sclerostin treatment delivered to MM-bearing mice was effective in increasing trabecular bone volumes by 46% and trabecular thickness by 30%, returning their bone volumes to similar levels of the non-tumour control mice and prevented further MBD. Two cell lines were used in this study, both exhibiting a positive effect, demonstrating the heterogeneity of anti-sclerostin treatment. Although mice with different immunodeficiency status were used for the two different cell lines, which may have contributed to the different results obtained, this work has now been further developed into three myeloma cell lines, which demonstrated that sclerostin is an osteocyte-specific protein and not released by myeloma cells [30]. Treatment of the myeloma-bearing mice in all three cell lines with an anti-sclerostin antibody caused an increase in osteoblastogenesis, reduced the development of osteolytic lesions and prevented myeloma-induced bone loss whilst increasing bone strength. Bone resorption was not prevented; however, combining anti-sclerostin treatment with the bisphosphonate, zoledronic acid, significantly improved bone strength compared to either treatment alone.

Eda et al. [69] further confirmed that mice injected with MM had higher levels of sclerostin and hypothesised that the decrease in β-catenin levels was the result of this. When treated with anti-sclerostin (scl-ab), trabecular bone thickness and volume increased in these mice. Also demonstrated was a potential link that Dkk-1 mediates the increase in sclerostin via inducing its release from osteoblasts.

Scl-ab has recently been shown to reduce bone marrow adipose tissue (BMAT) [75]. BMAT creates an optimal environment for MM by secreting growth factors such as IL-6, signalling molecules such as adipokines and fatty acids, creating an energy source and endocrine secretions that optimise MPCs’ growth and induce osteolytic lesions [75, 76]. BMAT differentiation is regulated by sclerostin, which inhibits Wnt signalling in pre-adipocytes and promotes adipogenesis [77]. Thus, inhibiting sclerostin reduces BMAT differentiation and increases bone formation.

Sclerostin is a promising target and its inhibition has been shown to be beneficial in postmenopausal women and osteoporosis; however, currently there are no clinical trials for MM [73, 78]. The potential for a dual target with Dkk-1 may also be a promising therapeutic in the future [72].

### Transforming Growth Factor Beta (TGF-β)

TGF-β is part of the TGF-β superfamily and has been implicated in various cancers for tumour-induced bone disease [6]. MBD causes an increased release of TGF-β by osteoclasts; however, the mechanism of TGF-β tumour-induced bone disease is unknown. A potential mechanism outlined by Balooch et al. [79] is that TGF-β activates SMAD3, which in turn binds to osteoblast promoters such as the transcription factor Runx2, sequentially suppressing the transcription of genes involved in osteoblast differentiation.

Nyman et al. [8] investigated the use of TGF-β inhibitor neutralising antibody (1D11) in myeloma-bearing mice. This improved the bone disease in mice and increased osteoblast differentiation. However, there was no improvement of overall tumour burden in these mice. There were differing results between the cell lines, which remain unexplained, and the long-term side effects were not explored such as widespread inflammation or cardiovascular defects, which would be an important result to establish in this treatment [80]. This is due to the dual action of TGF-β, as TGF-β can act as both an oncogene and a tumour suppressor [81]. Inhibiting the tumour-suppressing action of TGF-β may induce these side effects; however, these have yet to be confirmed in clinical trials [80].

Lu et al. [82] inhibited a different mechanism of TGF-β tumour-induced bone disease, which involves Thrombospondin1 (TSP-1). TSP-1 activates latent TGF-β that has been deposited by MPCs. A TGF-β inhibitor, SRI31277, was administered to mice with highly osteolytic lesions (human CAG-hpse cell lines) and showed a decrease in tumour burden and a decrease in phosphorylated SMAD2, which was associated with a decrease in osteoclasts and an increase in osteoblastogenesis. There were no noted side effects, which if translated into patients would be valuable. However, osteolytic lesions were only examined in one cell line, questioning how representative this would be in MM.

### Activin A and Sotatercept

Activin A is a member of the TGF-β superfamily alongside BMPs. Activin A is released from osteoblasts and osteoclast precursors and has been shown to be elevated in patients with MM. Oslen et al. [83] used in vitro models to establish that both TGF-β and BMPs share 3 receptors: activin receptor type 2A (ACVR2A), activin A receptor type 2B (ACVR2B) and activin receptor-like kinase-2 (ALK2). BMPs induce MPC death via these receptors and through activation of their downstream molecules SMAD 1/5/8. Activin A antagonises BMP-6 and BMP-9 by competing for their receptors ACVR2A/ACVR2B/ALK2 and therefore inhibit BMP-induced apoptosis of MPC [83, 84]. Furthermore, activin A activates RANK/RANKL to promote osteoclastogenesis and drives the process of osteolytic lesions.

Sotatercept is a soluble recombinant activin receptor type IIA (ActRIIA) ligand fused to the human FC-IG fragment and binds activin A/B plus members of the TGF-β superfamily to disrupt downstream cascades. Abdulkadyrov et al. [84] demonstrated during a phase II randomised control trial that sotatercept as an addition to melphalan, prednisolone and thalidomide caused an anabolic effect and increased the biomarker bone alkaline phosphatase (bALP), indicating improved bone turnover.

There are many limitations to this study however, including a small patient size with heavily weighted numbers in the intervention group and unclear side effect profile interactions between each drug and uncertainty if a significant change was actually seen when using sotatercept.

Currently, a clinical trial (NCT01562405) recruiting patients for the use of sotatercept in combination with lenalidomide or pomalidomide and dexamethasone is being undertaken. However, at present the evidence for the use of sotatercept is still to be determined.

### Agents that Combine Antitumour Activity and Bone Anabolic Effects

Proteasome inhibitors (PIs) inhibit the transcription factor NF-kB, thus reducing RANKL-mediated osteoclast differentiation, and also decrease the degradation of the NF-kB inhibitor I-kB, therefore preventing NF-kB from activating IL-6 and antiapoptotic genes. PIs synergistically produce an anabolic effect by increasing osteoblast differentiation through the upregulation of BMP-2 and the transcription factor Runx2 and reducing sclerostin levels [85, 86]. Initially, PIs have been used in combination with steroids, such as dexamethasone, and immunomodulatory agents, such as lenalidomide. Durie et al. [87] demonstrated an anti-myeloma effect of using the PI bortezomib, with 15.7% of patients having a complete response when treated with bortezomib compared to 8.4% of patients in the control group (lenalidomide and dexamethasone). There was an increase in adverse side effects when treated with bortezomib, including 33% of patients developing neurological toxic effects compared to 11% of patients in the control group.

Terpos et al. [86] have shown that bortezomib, even as a monotherapy, has anabolic activity, promoting osteoblastogenesis and leading to increased bone formation and bone mineral density in patients with relapsed/refractory MM. Harnessing these effects coupled with the potent anti-myeloma effects seen with proteasome inhibitors is a promising strategy requiring further evaluation [87]. However, Sezer et al. investigated bortezomib consolidation alone vs. observation alone on MM-related bone disease who had received frontline high-dose therapy and autologous stem cell transplantation and found that there was no difference between each group of patients [88]. However, there were multiple limitations to this study including that patients may have had prior bortezomib induction-based therapy, chemotherapy and BPs in patients, which may have influenced bone mineral density and bone metabolism markers.

Second-generation PIs such as carfilzomib have been approved for use in the UK when two other treatment plans have failed and has been shown to have a better side effect profile in regards to neuropathies, but unfortunately has a higher number of adverse effects in total, particularly in relation to cardiac events [89]. In 2015, the FDA approved the first oral PI, ixazomib, for those with refractory MM, which has the potential to overcome resistance and, in preclinical studies, has been shown to have a bone anabolic effect [90]. Both ixazomib and carfilzomib demonstrate bone anabolic effects similar to bortezomib and, coupled with their anti-myeloma effects, could be promising therapeutics [89, 90].

Epigenetic changes caused by MM play a role in MBD and disease progression. MM induces repressive epigenetic histone changes at the Runx2 locus by promoting the transcriptional repressor growth independent factor 1 (GFI1), which binds to Runx2, recruits histone modifiers such as histone deacetylase 1 (HDAC1) and suppresses Runx2 which is required for osteoblast differentiation [91]. Importantly, the recruitment of histone modifiers, such as HDAC1, is required to maintain the suppression of Runx2 [92]. Adamik et al. demonstrated that the inhibition of HDAC1 reversed the repression of Runx2 and increased osteoblast differentiation [92]. HDAC inhibitors (HDACi), such as vorinostat, could act as both an anabolic agent by increasing osteoblast differentiation and an anti-myeloma agent. HDACi decrease cell proliferation through reducing the viability of IL-6, induce cell cycle arrest at the G1/S phase and induce apoptosis of MPC via upregulation of both the intrinsic and extrinsic apoptotic pathways [93].

Although beyond the scope of this review, a plethora of new agents have been developed that are predominantly anti-myeloma chemotherapies but also have some positive effects on the regulation of MBD. These include the immunomodulatory compounds (thalidomide, lenalidomide, pomalidomide), monoclonal antibodies (daratumumab, elotuzumab) and histone deacetylase inhibitors (panobinostat) [1, 5]. There are also some novel agents in clinical trials including a kinesin spindle protein inhibitor, filanesib (Clinical Trial: NCT02384083), and an exportin 1 inhibitor, selinexor (Clinical Trial: NCT02336815), which have some promising preliminary results.

## Conclusion

MM survival outcomes and quality of life have dramatically improved with the introduction of many new encouraging agents. With patients surviving longer with their disease, this therefore highlights the need to introduce more effective agents for the treatment of MBD [7]. BPs remain the mainstay of treatment for MBD. However, their limited efficacy, inability to promote new bone formation and concerns over their side effect profile demonstrate the strong potential utility of bone anabolic agents. The mounting evidence of the benefits being exhibited by bone anabolic agents, such as anti-Dkk-1, anti-RANKL, anti-sclerostin and anti-TGF-β, does bring promise to improvements in the treatment of MBD.

However, further understanding of the multitude of factors involved in the pathophysiology of MBD and the complex interplay between MPCs and the BMME is essential, to truly determine the efficacy of these agents and their long-term outcomes.

With many agents in clinical trials and a plethora of factors to target, combination treatment presents the most potential for the management of MBD. The reduction in bone resorption coupled with new bone formation is necessary to decrease the burden of the disease. Bone anabolic agents in combination with both antiresorptive agents and anti-myeloma therapies may pave the way for future treatment of MBD, but further research is warranted to validate these outcomes for patients and ultimately determine their quality of life and survival.
